# A segmentation-combination data augmentation strategy and dual attention mechanism for accurate Chinese herbal medicine microscopic identification

**DOI:** 10.3389/fpls.2024.1442968

**Published:** 2024-11-29

**Authors:** Xiaoying Zhu, Guangyao Pang, Xi He, Yue Chen, Zhenming Yu

**Affiliations:** ^1^ Guangxi Colleges and Universities Key Laboratory of Intelligent Software, Wuzhou University, Wuzhou, China; ^2^ Guangxi Key Laboratory of Machine Vision and Intelligent Control, Wuzhou University, Wuzhou, China

**Keywords:** Chinese herbal medicine, deep learning, attention mechanism, cell recognition, data augmentation

## Abstract

**Introduction:**

Chinese Herbal Medicine (CHM), with its deep-rooted history and increasing global recognition, encounters significant challenges in automation for microscopic identification. These challenges stem from limitations in traditional microscopic methods, scarcity of publicly accessible datasets, imbalanced class distributions, and issues with small, unevenly distributed, incomplete, or blurred features in microscopic images.

**Methods:**

To address these challenges, this study proposes a novel deep learning-based approach for Chinese Herbal Medicine Microscopic Identification (CHMMI). A segmentation-combination data augmentation strategy is employed to expand and balance datasets, capturing comprehensive feature sets. Additionally, a shallow-deep dual attention module enhances the model's ability to focus on relevant features across different layers. Multi-scale inference is integrated to process features at various scales effectively, improving the accuracy of object detection and identification.

**Results:**

The CHMMI approach achieved an Average Precision (AP) of 0.841, a mean Average Precision at IoU=.50 (mAP@.5) of 0.887, a mean Average Precision at IoU from .50 to .95 (mAP@.5:.95) of 0.551, and a Matthews Correlation Coefficient of 0.898. These results demonstrate superior performance compared to state-of-the-art methods, including YOLOv5, SSD, Faster R-CNN, and ResNet.

**Discussion:**

The proposed CHMMI approach addresses key limitations of traditional methods, offering a robust solution for automating CHM microscopic identification. Its high accuracy and effective feature processing capabilities underscore its potential to modernize and support the growth of the CHM industry.

## 1 Introduction

Chinese Herbal Medicine (CHM) is a cornerstone of traditional Eastern healthcare and has been integrated into disease treatment. With roots deeply embedded in ancient Chinese science, CHM symbolizes Eastern medicine’s cultural heritage and underscores a comprehensive medical paradigm that has garnered global recognition for its efficacy. This acknowledgement has notably surged during the COVID-19 pandemic, highlighting the potential of CHM in contributing to contemporary medical practices and prompting a broader international acceptance and trust in its remedies. The burgeoning trust in CHM has catalyzed a substantial expansion of its market, with recent data indicating an annual output reaching 4,555 million tons and daily testing frequencies surpassing 22 million instances. CHM includes plant, animal, and mineral medicines, and according to the Chinese Materia Medica, there are 8,980 kinds of herbs in total. With the addition of medicines used by ethnic minorities, the number of varieties has reached more than 28,000 so far (Li, 1999). These figures reflect the growing reliance on CHM for healthcare purposes and underscore the potential of the fast inspection market within this domain. However, the predominant methodologies employed for CHM identification, particularly through traditional manual microscopy, present numerous challenges. These methods are labor-intensive, require extensive expert knowledge, suffer from low throughput due to the microscopic equipment’s limited field of view, and are prone to human error from tester fatigue, potentially leading to misjudgments.

There are four traditional identification methods for CHM: original plant (i.e., animal) identification, character identification, microscopic identification, and physical and chemical identification. Original plant (i.e., animal) identification Yin et al. (2019) was performed by observing the appearance of plants, animals, and minerals in morphological form and classifying herbs using knowledge of taxonomy. Character identification Thongkhao et al. (2020) was carried out by eyes, hand, nose, mouth taste, water test, fire test, and other simple ways to identify medicinal materials. Microscopic identification Ichim et al. (2020) uses microscopy to observe tissue structure, cell shape, and the features of inclusions of medicinal herbs to determine the nature of cell walls and cell inclusions or the distribution of active ingredients of certain species in tissues, and finally to achieve the identification of authenticity of herbal medicines. Physical and chemical identification Peng and Tsa (2020) is to use certain physical, chemical, or instrumental analysis methods to identify the authenticity, purity, and quality of traditional Chinese medicines. Generally, the first three conventional identification techniques rely primarily on abundant working experience, making distinguishing similar or analogous substances difficult.

However, physical and chemical identification is a highly advanced technique, particularly tedious, requiring specialized equipment and high costs. The need for an advanced, reliable, and less subjective method is evident, particularly to keep pace with the increasing scale of CHM testing and support the industry’s growth and modernization efforts.

**Figure 1:**
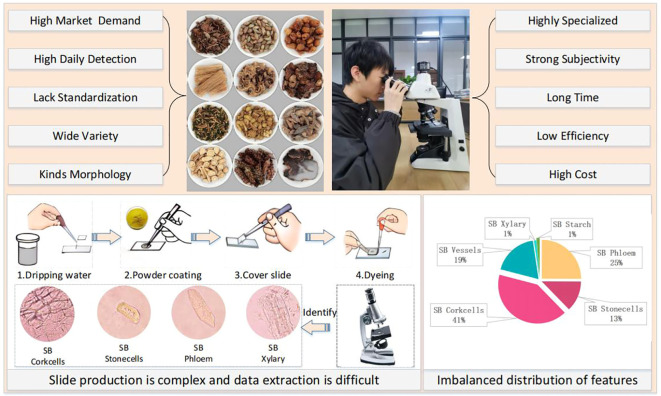
Challenges in Chinese herbal medicine microscopy identification.

The development of artificial neural networks has opened up new avenues for image recognition, and deep learning-based methods have shown great success in various applications Chen et al. (2022); Jiang et al. (2022). As shown in **Figure 1**, several key challenges hinder the development of automated CHM microscopic identification systems:

1) **Data collection difficulties and class imbalance:** We found no publicly available herbal microscopic image datasets after reviewing the literature and searching search engines. CHM image datasets often exhibit significant class imbalance, where certain cell types or features are underrepresented. This can lead to biased models that perform poorly on rare classes.

2) **Small and Uneven Features:** CHM microscopic images contain small and unevenly distributed features, making it difficult for traditional object detection algorithms to locate and classify them accurately.

3) **Incomplete and Blurriness Cell Structures:** The grinding process used to prepare CHM samples can damage cell structures, resulting in incomplete or ambiguous features that further complicate identification.

This paper proposes a novel methodology, CHMMI, which innovatively applies a segmentation-combination method for data augmentation, allowing the model to capture more comprehensive feature sets from the available microscopic images. Furthermore, by integrating attention mechanisms, CHMMI enhances the model’s focus on relevant features across different layers, thereby improving the accuracy of CHM identification. Finally, features across multiple scales and dimensions effectively detect and identify herbal microscopic images. The contributions of this paper can be summarized as follows:

- We propose a data augmentation strategy for generating more datasets by random cutting and random combination for the problem that a single image in CHM micrographs includes many different cells, which can extend and balance the datasets and provide a solid foundation for the training and prediction of the actual model.
- We develop a shallow-deep dual attention module that effectively captures valid auxiliary information from different channels in shallow and deep layers. This facilitates the processing of small, uneven features and incomplete and blurry cell structures in CHM.
- In the final prediction stage, we integrate three features with different object scales through a multi-scale inference module to predict objects in the image.
- We evaluate the performance of CHMMI through a series of comparison experiments with existing state-of-the-art approaches, such as YOLOv5 Zhu et al. (2021), SSD Liu et al. (2016), Faster R-CNN Khan et al. (2022), and ResNet He et al. (2016). The experimental results demonstrate that CHMMI achieves higher accuracy than these approaches, highlighting its potential for practical application in CHM microscopic identification.

## 2 Related work

Image recognition has significantly advanced by integrating deep learning techniques, predominantly categorized into one-stage and two-stage detection algorithms. These methodologies have been extensively employed across various sectors, including healthcare, autonomous driving, and precision agriculture, progressively encompassing microscopic image analysis for CHM.

### 2.1 Deep learning-based image recognition methods

Several image recognition approaches based on deep learning have been proposed, including two-stage detection algorithms (e.g., Faster RCNN, SSD) and one-stage detection algorithms (e.g., RetinaNet, YOLO). These algorithms have achieved state-of-the-art performance in various image recognition tasks, such as face detection, object detection, and image classification. For example, Sun et al. (2018) improved the state-of-the-art Faster RCNN framework by combining several strategies, proposed a new face detection scheme using Deep Learning, and achieved the state-of-the-art detection performance on the well-known FDDB face detection benchmark evaluation. Zhai et al. (2020) proposed an improved SSD object detection algorithm based on Dense Convolutional Network (DenseNet) and feature fusion; the algorithm replaces the original backbone network VGG-16 of SSD with DenseNet-S-32-1 to enhance the feature extraction ability of the model. Wang et al. (2020) proposed an automatic ship detection model based on RetinaNet, the model solves the problem that ships have multi-scale shape features in SAR images due to the diversity of SAR imaging patterns and the diversity of ship shapes, resulting in poor recognition rates. Yu et al. (2021) proposed a Deep Learning model named YOLOv4-FPM to realize real-time detection for bridge cracks by unmanned aerial vehicles. Yan et al. (2021) proposed an improved yolov5-based lightweight apple target detection approach for apple picking robots to address the problem that existing apple detection algorithms cannot distinguish between apples obscured by tree branches and apples obscured by other apples, leading to picking failure. Kim et al. (2022) proposed an approach with Maritime Dataset on modified YOLO-V5 with the SMD-Plus, the approach solves the problem of poor recognition rates due to the presence of noisy labels and imprecisely positioned bounding boxes in SMD.

The YOLO series of algorithms have been widely used in various applications, including object detection, pedestrian detection, and facial recognition. YOLOv5, in particular, has been shown to be effective in detecting objects in images with varying sizes, scales, and orientations.

### 2.2 Microscopic image recognition for Chinese herbal medicine

In microscopic image recognition for CHM, researchers focus on several challenges, including the uneven distribution of sample classes and small differences between classes, stereoscopic features of cells, and the effect of background color on recognition rate.

For the first type of problem, Wang et al. (2020b) used techniques such as dynamic ReLU function and multi-channel color space to use Xception with obvious classification effect as the base network, and replaced the static ReLU in the network with dynamic ReLU so that each small sample has a unique ReLU parameter. For the second type of problem, Ying et al. (2012) analyzed the differences in the characteristics of cross-sections and powders of stems and leaves of two herbs, Buddleja albiflora Hemsl and Buddleja davidii Franch, which provided important criteria for the recognition of these two herbs. Ye et al. (2014) used a method of fusion of coaxial X-ray and micro-CT imaging techniques for three-dimensional nondestructive *in situ* microscopic imaging of the microscopic image of Amomi Rotundus Fructus and Alpiniae Katsumadai Semen seeds. This method obtained information on the microscopic image’s internal microstructure and different cross-sectional orientations. For the third type of problem, Wang et al. (2017) used MATLAB software to program the stitching of the cross-sectional tissue images of the CHM Achyranthes bidentata and Cyathula officinalis. The features such as texture, color, and invariant moment of the microscopic image were extracted to recognize the two herbs effectively. Wang et al. (2020a) used a multi-channel and improved attention method to stitch the microscopic image data of 34 herbal catheters with images of different color spaces of the images themselves before inputting them into the network, and the method effectively improved the accuracy of recognition.

The above work mainly focuses on researching a single problem. However, three types of problems simultaneously exist in detecting CHM microscopic images. Our CHMMI method shows promising results.

## 3 Problem statement

**Figure 2:**
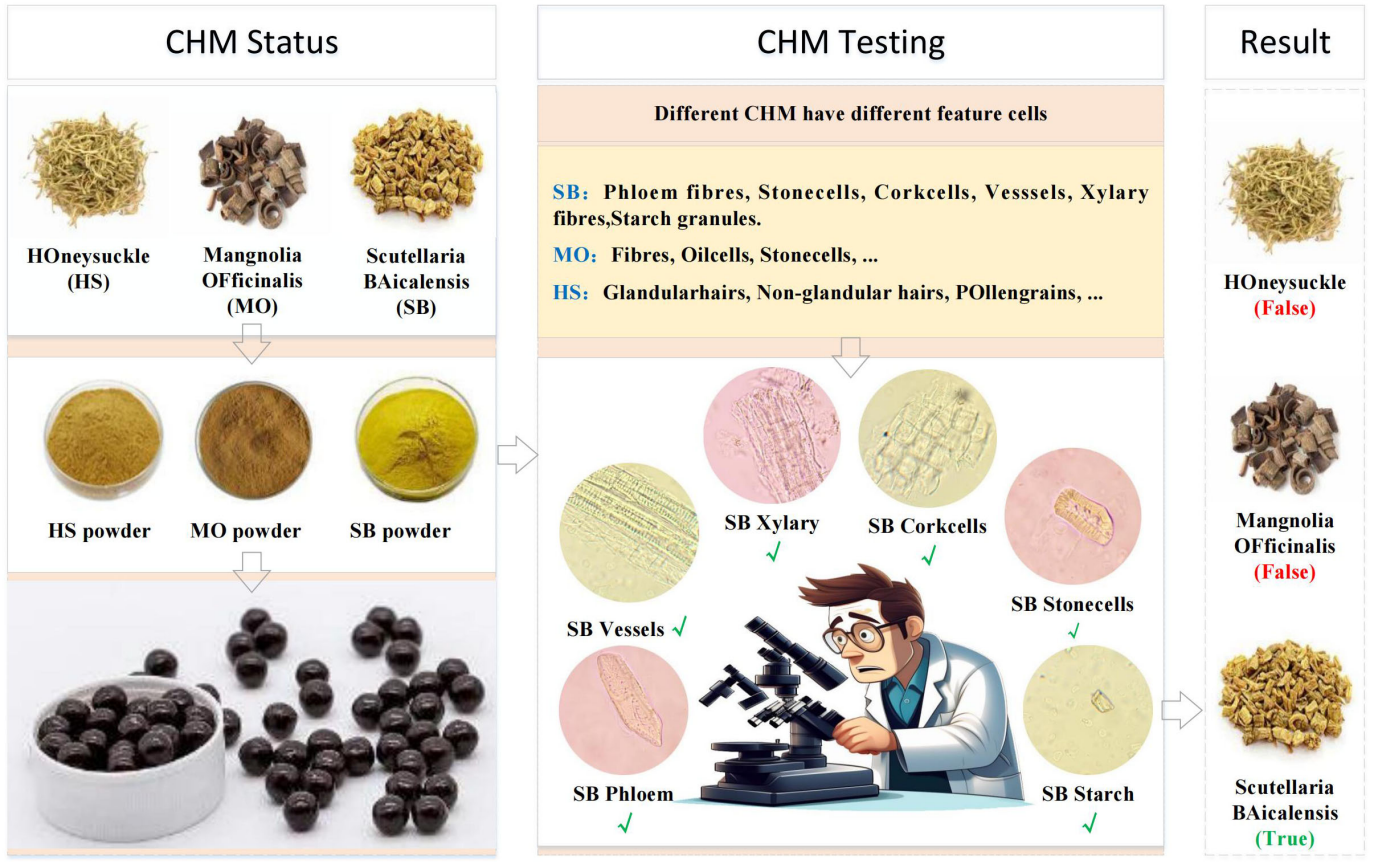
Quality testing process of herbal medicine by feature cells.

CHM identification relies on the microscopic examination of herbal powders to verify their authenticity. Each herb can be identified by specific cellular structures, termed “feature cells”, as illustrated in **Figure 2**. For example, identifying *Scutellaria baicalensis* requires detecting six distinct feature cells in microscopic images. We believe that the features of herbal microscopic images have a direct relationship with the accuracy of cell recognition. Therefore, we formulate the problem: How can we achieve automated herbal microscopic identification on an insufficient data-level scale and with an unbalanced distribution of sample data?

To systematically approach the problem, we define the terms and notations used in this study: Given the dataset of microscopic images *X* and their corresponding annotations *Y*, the objective is to develop a fitted model f(X) that accurately identifies and classifies the feature cells in new, unseen microscopic images of CHM.

Let $\text{X}=\;\left\{{\text{X}}_{1},{\text{X}}_{2},\ldots ,{\text{X}}_{\text{i}},\ldots ,{\text{X}}_{\text{N}}\right\}$ represents the set of microscopic images used in the dataset, where each image $X_{i}$ may contain one or more cell features and *N* is the total number of images. Associated with each image are target bounding boxes $\text{Y}=\;\left\{{\text{Y}}_{1},{\text{Y}}_{2},\ldots ,{\text{Y}}_{\text{i}},\ldots ,{\text{Y}}_{\text{N}}\right\}$ , where each $Y_{i}$ contains one or more bounding boxes indicating the location of feature cells within the image *X_i_*. For each feature cell *j* in image $X_{i}$ , the bounding box is represented as $Y_{i}^{j}=\left\{\left[x_{i1}^{j},y_{i1}^{j}\right],\left[x_{i2}^{j},y_{i2}^{j}\right]\right\}$ and $\left[x_{i1}^{j},y_{i1}^{j}\right]$ are the coordinates of the upper-left and lower-right corners of the bounding box, respectively.

## 4 Methods

**Figure 3:**
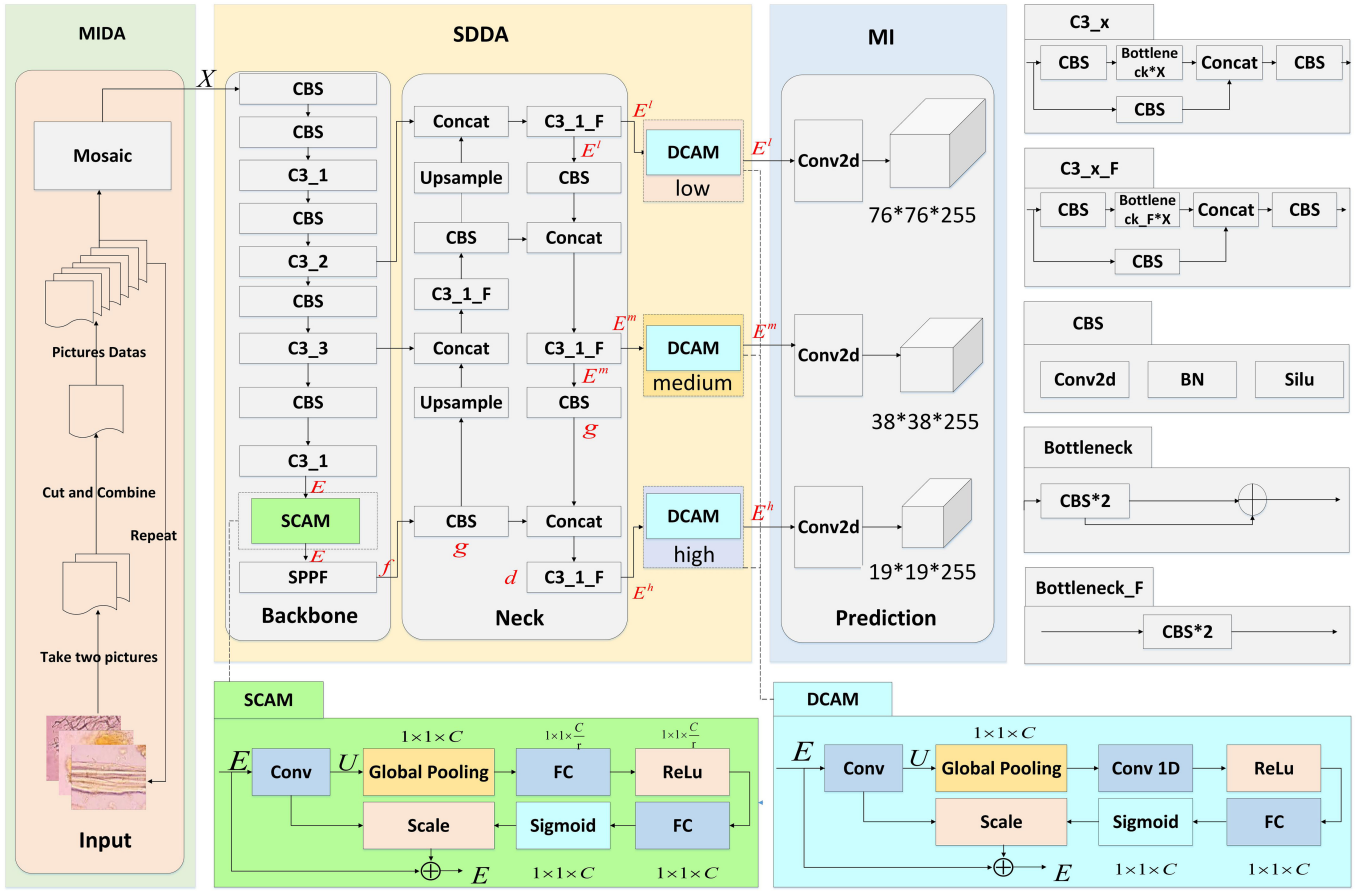
Network structure of CHMMI. MIDA is allowed to expand and balance the existing herbal microscopic image dataset. SDDA better captures cell features in the microscopic examination of CHM cells. MI integrates and analyzes features across multiple scales and dimensions intelligently to make final decisions.

This section presents three main modules: the Microscopic Image Data Augmentation (MIDA) Module, the Shallow-Deep Dual Attention (SDDA) Module, and the Multi-scale Inference (MI) Module, as shown in **Figure 3**. These modules are designed to improve the accuracy and reliability of microscopic image analysis in the study of Chinese herbal medicine.

### 4.1 Microscopic image data augmentation module

**Figure 4:**
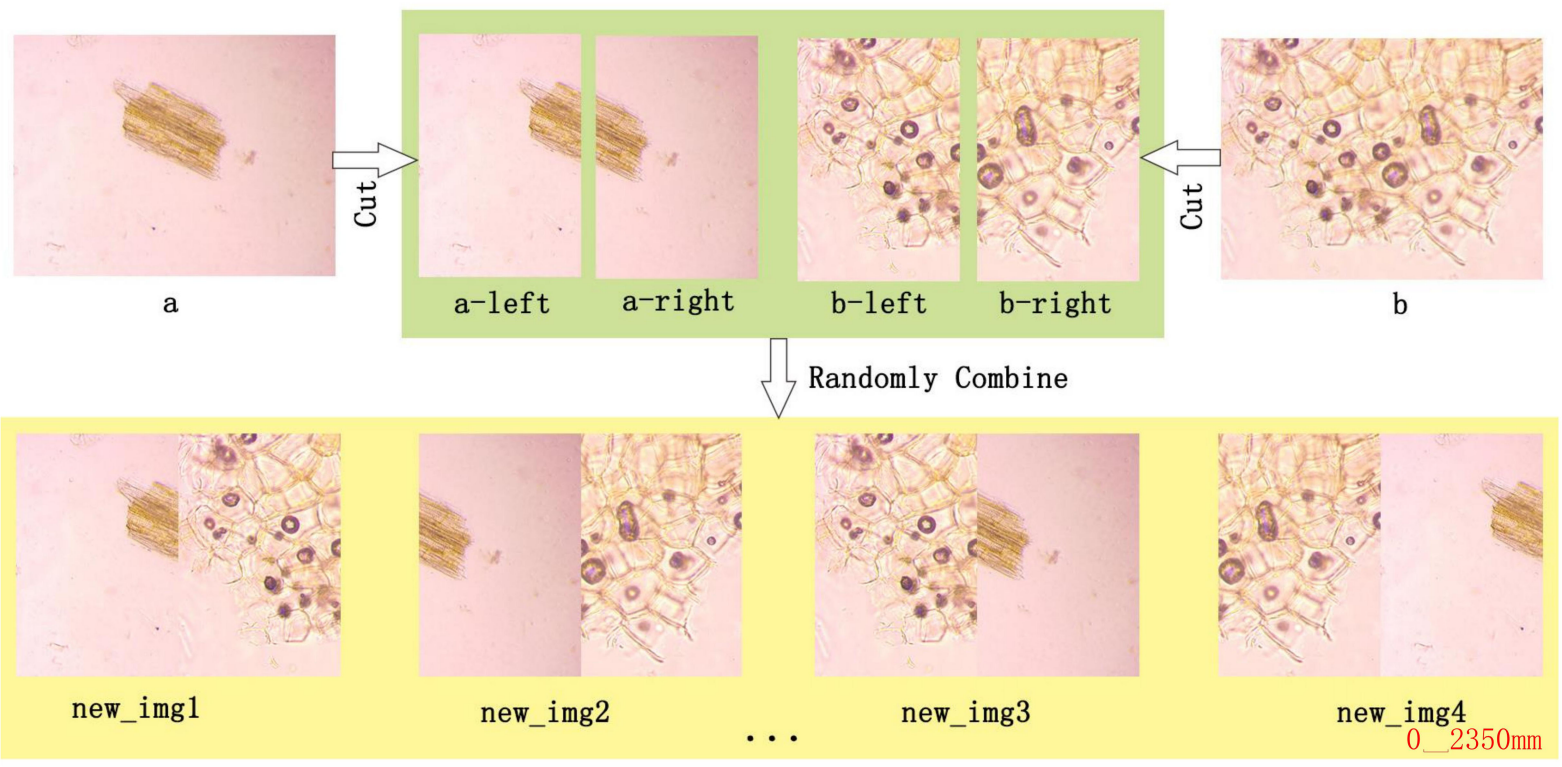
Example of MIDA processing. The MIDA module enhances the dataset through image segmentation **(A)** into a-left and a-right, **(B)** into b-left and b-right) and recombination (new_img1, new_img2, new_img3, new_img4, etc.), multiplying the number of images and introducing variability in the dataset.

The MIDA module is used to augment and balance the available dataset for training and predicting herbal microscopic images. Our MIDA module associates some of the images with features that are only partially or partially clear, enhancing the representation of specific cell types or features. The detailed steps of MIDA are listed as follows:

1. **Random Selection:** Randomly select two images from the original dataset, such as **Figures 4A, B**.
2. **Horizontal Segmentation:** Each image is segmented into two halves along the horizontal axis.
3. **Recombination:** Two distinct segments are chosen and stitched together to form four new images from the pool of segmented halves. This ensures that the resultant image differs from the original images (a) and (b), thus enhancing feature representation and diversity.
4. **Augmentation Techniques:** Beyond simple recombination, MIDA incorporates advanced image processing techniques inspired by YOLOv5, such as mirroring, translation, and rotation. These techniques enhance the dataset’s diversity further, enabling the model to generalize better across unseen images during inference.

### 4.2 Shallow-deep dual attention module

The SDDA module addresses several prevalent issues in the microscopic examination of CHM cells, such as the uneven distribution of cells with distinct morphological features and incomplete and blurry cell structures. This module integrates two attention mechanisms: the Shallow Channel Attention Mechanism (SCAM) and the Deep Channel Attention Mechanism (DCAM).

#### 4.2.1 Shallow channel attention mechanism

The core concept of SCAM is to address the problem of uneven cell distribution in CHM cell images by assigning more weights to cell information with significant morphological features while ignoring unimportant feature information, thus improving the image feature recognition rate. The SCAM mechanism consists of three main components: Squeeze, Excitation, and Scale, as shown in **Figure 3A**. The Squeeze operation performs a global average pooling on the image features to compress the features and reduce the dimensionality. The Excitation operation predicts the importance of each channel using a gating mechanism of the Sigmoid form, which enables the network model to learn the importance of each channel automatically. Finally, the Scale operation outputs the resulting 1 × 1 × *C* real numbers with the original feature images, where *C* is the number of channels. The specific implementation of the SCAM module is given as follows:

Firstly, the input *E* is transformed through a series of convolution operations to obtain the features *U*. Use $V=\;\left[v_{1},v_{2},\ldots ,v_{C}\right]$ to denote a series of convolution kernels, where $v_{C}$ denotes the parameters of the *c*th layer convolution. That is, the output feature $\text{U}=\;\left[{\text{u}}_{1},{\text{u}}_{2},\ldots ,{\text{u}}_{\text{C}}\right]$ can be expressed as follows:


(1) $$u_{c}=v_{C}*E=\sum_{S=1}^{C^{\prime }}\;V_{C}^{S}*E^{S}$$

where ∗ denotes the convolution operation $V_{C}^{S}$ denotes the *c*th convolution kernel of the *s*th input, $E^{S}$ denotes the *s*th input.

Secondly, a global average pooling Zaidi et al. (2022) is performed by the Squeeze operation in the SCAM module for the image features *U*, intending to compress the image features *U*. The compressed image feature becomes a one-dimensional real number *z*, and *z* is denoted as the residual channel statistic. Suppose the length of the output is set to *c*, $Z_{c}=\left[z_{1},z_{2}\ldots \ldots ,z_{c}\right]$ , (x,y) denotes the size is the feature of W∗H , *x* is the horizontal coordinate and *y* is the vertical coordinate. That is, the *c*th element of *z* can be given by is expressed as:


(2) $$z_{c}=\frac{1}{H\times W}u_{c}\left(x,y\right)$$

Immediately after, the importance of each channel is predicted by the Excitation operation in the SCAM module using a gating mechanism of the Sigmoid form to obtain the nonlinear relationship between the different channels. Assuming that $W_{1}\;\in \;R^{\frac{r\times c}{c}},\;W_{2}\;\in \;R^{\frac{r}{c\times c}}$ are two different fully connected layers, *r* is the dimensionality reduction rate when *r* is small, the global information of the upper layer can be better preserved, but the computational cost will be relatively increased. To balance propagation speed and detection accuracy, refer to SENet Wang and Yoon (2021) and set *r* to 16. The final output parameter of the Excitation operation is the weight *ω* of each feature channel, and *ω* can be expressed as follows:


(3) $$\omega =\sigma \left(W_{2}\times \delta \left(W_{1}\times z\right)\right)$$

where, *σ* is the Sigmoid function, *δ* is the ReLU activation function.

Finally, the resulting 1 × 1 × *C* real numbers are output with the original feature images by the Scale operation in the SCAM module. The formula is listed as follows:


(4) $${\tilde{E}}_{C}=\omega_{c}u_{c}$$

where ${\tilde{E}}_{C}=\left[{\tilde{e}}_{1},{\tilde{e}}_{2},\ldots ,{\tilde{e}}_{c}\right]$ denotes the product of the corresponding pixel points in the channel between the image feature $u_{c}\in R^{W\times H}$ and the scalar $\omega_{c}$ . The Scale operation enables the network model to automatically learn the importance of each channel, thus enhancing the recognition of image features.

#### 4.2.2 Deep channel attention mechanism

The DCAM module subtly enhances the feature representation extracted from the cells by adaptively recalibrating the channel feature response to address the CHM’s incomplete and blurriness cell structure. The core of DCAM lies in the clever use of the ECA attention mechanism to function at deeper layers of the network, especially at the level where the semantic information is becoming progressively more abstract and where information localization is critical in accuracy. This is particularly beneficial in the context of the CHMMI network structure, where the fusion of features across different dimensions is critical for achieving high detection performance.

In the CHMMI network, the DCAM is strategically positioned within the ‘Neck’ layer, a critical juncture for feature fusion and refinement. This layer utilizes architectures like the Path Aggregation Network (PAN) and Feature Pyramid Network (FPN) to effectively amalgamate rich locational details from shallow layers with deeper, semantically strong features. The goal is to enhance the upward and lateral flow of information across the network, ensuring that each level receives a balanced mix of depth-specific features. The specific implementation of the DCAM module is given as follows:

The Neck layer has three different dimensional feature outputs towards the Prediction layer, namely low $\left(E^{l}\right)$ , medium $\left(E^{m}\right)$ , and high $\left(E^{h}\right)$ . Taking $E^{h}$ as an example, $E^{h}$ can be expressed as follows:


(5) $$E^{h}=\tilde{(E}+f+g)⊕\left(E^{m}+g\right)+d$$

where + denotes the serial processing of features. ⊕ denotes tensor stitching, assigning weights to the input features at different levels. *f* denotes the processing of input features by the SPPF module, *g* denotes the processing of input features by the CBS module, and *d* denotes the processing of input features by the C3_1_F Zhu et al. (2021) module.

The DCAM module modifies the conventional channel attention by implementing a three-step process—Squeeze, Convolve, and Scale—tailored to handle multi-dimensional data more effectively:

Firstly, the input $E^{h}$ is transformed through a series of convolution operations to obtain the feature $U^{h}$ .

Secondly, the global average pooling of the feature $U^{h}$ is performed using the Squeeze operation to compress the feature $U^{h}$ . The feature $U^{h}$ is compressed into a one-dimensional real number *z*. For the *c*th cell in *z*, the following is calculated:


(6) $$z_{c}=\frac{1}{H\times W}u_{c}^{h}\left(x,y\right)$$

Next, to avoid dimensionality reduction, the DCAM module is implemented by a one-dimensional convolution with a convolution kernel size of *k* cross-channel information interaction. The equation is expressed as follows.


(7) $$\omega =\sigma \left(C1D_{k}\left({\text{z}}_{\text{c}}\right)\right)$$

where, *C*1*D* is the one-dimensional convolution Wang et al. (2019). *k* is the size of the one-dimensional convolution kernel to represent the cross-channel range of interactions. *k* has a feature mapping relationship with the number of channels *c*, which can be calculated adaptively by the following equation.


(8) $$k=\psi \left(C\right)=\|\log_{2}(C)/\gamma +b/\gamma {\|}_{odd}$$

where, $∥n{∥}_{odd}$ is the closest odd number to *n*. Referring to the experiments in the literature ECA Wang et al. (2019), *γ*  and *b* are set to 2 and 1. By mapping *ψ*, high-dimensional channels have longer interactions, while low-dimensional channels have shorter interactions using nonlinear mappings.

Lastly, the obtained weights and the original feature image are output by the Scale operation in DCAM, and the final residual features are represented as follows.


(9) $${\tilde{E}}_{C}^{h}=\omega_{c}.u_{c}^{h}$$

Similarly, the low-dimensional residual features ${\tilde{E}}_{C}^{l}$ and the medium-dimensional residual features ${\tilde{E}}_{C}^{m}$ can be obtained

### 4.3 Multi-scale inference module

The MI module is a crucial component of the CHMMI network and is responsible for effectively detecting and identifying herbal microscopic images. It intelligently integrates and analyzes features across multiple scales and dimensions, enabling the model to capture local and global information from the input images. The module consists of two main components: feature fusion and microscopic recognition.

The feature fusion module integrates features from different scales and channels using a feature pyramid network (FPN), allowing the model to capture local and global information from the input images. This is achieved by up-sampling the feature maps and fusing them with the shallow feature maps, resulting in a richer feature representation that facilitates accurate identification of cellular structures.

The microscopic recognition module is responsible for predicting the presence and location of cellular features in the input images. This is accomplished by applying a combination of convolutional and spatial attention mechanisms to focus on relevant regions of the images. The module outputs a set of bounding boxes and confidence scores for each predicted feature. The input herbal microscopic images are meshed, and if there is a center of the object in the mesh, the mesh is used to predict this object. The prediction of each grid cell includes information on the location of the three object-bounding boxes and a confidence level. An object box corresponds to four position information (x,y,w,h) and one confidence information. Where *x* and *y* denote the location of the object’s center point, *w* and *h* denote the center point’s width and height from the object’s two sides. Confidence C represents the predicted object box contains two-fold information about the confidence of the object and the accuracy of the prediction of this object box, and the formula is expressed as follows:


(10) $$\text{C}={\text{P}}_{\text{r}}\left(obj\right)\times IOU_{B}^{A}$$

where IOU= (A∩B)/(A∪B) A denotes the real box, B denotes the predicted box, $IOU_{B}^{A}$ denotes the intersection ratio of A and B. when ${\text{P}}_{\text{r}}\left(obj\right)=1$ , it indicates that there is an object in the image, when ${\text{P}}_{\text{r}}\left(obj\right)=0$ , it indicates that there is no object in the image.

We use Non-maximum Suppression (NMS) Wu et al. (2020) to eliminate redundant prediction boxes and filter out high-quality detection results.

### 4.4 Training strategy

During the training phase, a three-part loss function is used: object loss, category loss, and confidence loss.

The object loss measures the difference between the predicted and ground-truth bounding boxes. It is calculated using the following equation:


(11) $$\begin{array}{l} l_{obj}=\sum_{i=0}^{S\times S}\sum_{j=0}^{N}I_{ij}^{obj}\left[{\left(x_{i}-{\hat{x}}_{i}\right)}^{2}+\;{\left(y_{i}-{\hat{y}}_{i}\right)}^{2}\right]\\ +\sum_{i=0}^{S\times S}\sum_{j=0}^{n}I_{ij}^{obj}\left[{\left(\sqrt{w_{i}}-\sqrt{{\hat{w}}_{i}}\right)}^{2}+\;{\left(\sqrt{h_{i}}-\sqrt{{\hat{h}}_{i}}\right)}^{2}\right] \end{array}$$

where *S* × *S* denotes the partitioning of the input image into *S* × *S* mesh grids; *N* denotes a grid responsible for predicting number of boxes; $\left(x_{i},y_{i},w_{i},h_{i}\right)$ denotes the position information of the real box; $\left({\hat{x}}_{i},{\hat{y}}_{i},{\hat{w}}_{i},{\hat{h}}_{i}\right)$ denotes the position information of the predicted box; $I_{ij}^{obj}$ denotes that the *j*th prediction box of each of the *i*th network is responsible for predicting object obj is 1, otherwise is 0.

The category loss measures the difference between the predicted class probabilities and the ground-truth class labels. It is calculated using the following equation:


(12) $$l_{cls}=\sum_{i=0}^{S\times S}\;I_{ij}^{obj}\sum_{c\in classes}({\left(p_{i}\left(c\right)-{\hat{p}}_{i}\left(c\right)\right)}^{2}$$

where, *c* denotes the number of categories; *p_i_* (*c*) denotes the probability of the true category; ${\hat{p}}_{i}\left(c\right)$ denotes the probability of the predicted category.

The confidence loss was calculated using CIOU Zheng et al. (2020), and the equation was expressed as follows:


(13) $$l_{ciou}=\sum_{i=0}^{S\times S}\;\sum_{j=0}^{n}\;I_{ij}^{obj}{\left(C_{i}-{\hat{C}}_{i}\right)}^{2}+\lambda_{noobj}\sum_{i=0}^{S\times S}\;\sum_{j=0}^{n}\;I_{ij}^{noobj}{\left(C_{i}-{\hat{C}}_{i}\right)}^{2}$$

where $I_{ij}^{noobj}$ denotes 0 when the *j*th prediction box of the *i*th network is not responsible for predicting an object and 1 otherwise. $\lambda_{noobj}$ is to reduce the confidence loss of the prediction box for the non-existent object obj. In this paper, reference paper Wang et al. (2021) sets $\lambda_{noobj}$ to 0.5.

The total loss is the weighted sum of the three components of object loss, category loss, and confidence loss, expressed by the following equation.


(14) $$L=\alpha l_{obj}+\beta l_{cls}+\gamma l_{ciou}$$

where, *α*, *β*, *γ* denote the weights of the three loss components respectively.

## 5 Experiments

To evaluate the performance of the proposed CHMMI method for microscopic image analysis of Chinese herbal medicines, we conducted a series of comprehensive experiments using our custom-built dataset. The experiments were designed to assess the effectiveness of CHMMI for accurately identifying and classifying different types of feature cells presented in the microscopic images of Scutellaria Baicalensis(SB) and Magnolia Officinalis(MO).

### 5.1 Experiment setup

#### 5.1.1 Datasets

**Figure 5:**
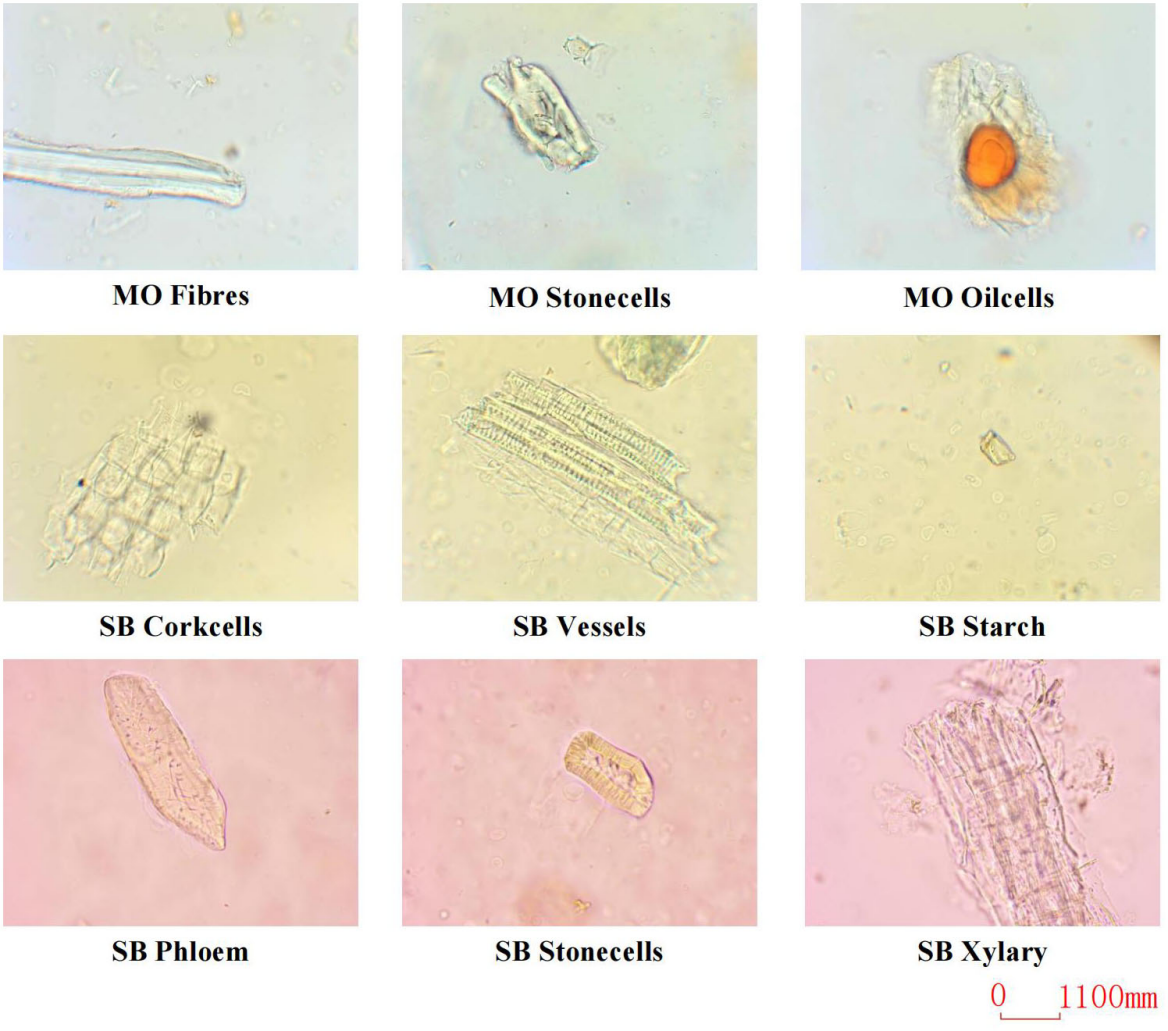
Sample images of the 9 cell types.

Due to the lack of publicly available datasets for microscopic images of Chinese herbal medicines, we constructed our dataset by preparing slides of powdered SB and MO. We used a Nikon E200 electron microscope with a 40/0.65 objective and the software Labeling to label the microscopic image of Chinese medicine feature cells. The resulting dataset consists of 11,060 microscopic images containing 12,840 labeled instances of nine distinct types of feature cells. The distribution of images and labeled instances for each feature cell type is shown in **Table 1**. These feature cells include Fibers, Stone cells, and Oil cells for MO, Phloem fibers, Stone cells, Corkcells, Vessels, Xylary fibers, and Starch granules for SB. **Figure 5** presents sample images of the nine feature cell types.

**Table 1:** Statistics of Chinese medicine microscopic image annotation dataset.

Dateset	MO			SB					
	Fibers	Stonecells	Oilcells	Phloem	Stonecells	Corkcells	Vessels	Xylary	Starch
**Images**	7555	1662	576	304	156	550	229	13	15
**Boxes**	9080	1726	644	353	171	580	257	13	16
**Images Total**	9793			1267					
**Boxes Total**	11450			1390					

To ensure a robust evaluation of the proposed CHMMI method, the dataset was partitioned into training and test sets following an 8:2 ratio. Furthermore, to rigorously assess the effectiveness of the CHMMI method and its individual components, we conducted a five-fold cross-validation experiment on the training dataset. This involved splitting the training data into five non-overlapping subsets. Each subset was then used in turn as a validation set while the remaining four subsets were combined for training. Applying each of the five trained models to the test set, generating five sets of prediction results for every test sample. Implementing a voting mechanism across the five predictions to determine the final predicted label for each test sample.

#### 5.1.2 Implementation details

We implemented the CHMMI method based on the PyTorch deep learning framework YOLOv5, training the model on an NVIDIA GeForce RTX 3090 GPU with 24GB memory. The model has trained 100 epochs with the Adam optimizer, using a learning rate of 0.001 and a batch size of 16. In our implementation, we adopted a three-scale anchor system: P3/8, P4/16, and P5/32. Specifically, the P3/8 scale anchors are designed to detect small targets, the P4/16 anchors are geared towards medium-sized targets, and the P5/32 anchors aim to detect large targets. This hierarchical structure ensures comprehensive coverage of the target size spectrum within the microscopic images.

#### 5.1.3 Evaluation metrics

To evaluate the CHMMI algorithm’s performance comprehensively, we select four evaluation metrics: precision, Recall, Average Precision (AP) curve, Mean Average Precision (MAP), and Matthews Correlation Coefficient(MCC). These metrics evaluate the algorithm’s ability to accurately identify and classify the feature cells present in microscopic images.

Precision denotes the ratio of true positive cases predicted to be true to all predicted positive cases Liu et al. (2018). It is calculated as:


(15) precision=TP/(TP+FP)

where TP denotes that the predicted value is the same as the true value, and the predicted value is a positive sample; FP denotes that the predicted value is different from the true value, and the predicted value is a positive sample.

Recall denotes the ratio of true positive cases predicted to be true to all true positive cases. It is calculated as:


(16) recall=TP/(TP+FN)

where FN denotes that the predicted value is not the same as the true value and the predicted value is a negative sample.

The AP curve is the area surrounded by the curve in two dimensions: Precision and Recall. Usually, Precision is higher when Recall is lower and lower when Recall is higher. That is, the larger the AP curve, the better the model’s performance.

MAP is a comprehensive evaluation metric focusing on sequence weights. It has become one of the most important practical metrics for image recognition problems in recent years. mAP@.5 indicates that the average AP of all images under each category is calculated at IoU=0.5, and the higher the value of mAP, the better the model’s performance.

MCC is an effective and comprehensive evaluation metric widely used in tasks with unbalanced sample categories, such as defect detection. It is particularly suitable for performance evaluation of binary classification models because it integrates the predictions of the model’s TP, TN, FP, and FN and is thus more robust than other metrics in evaluating the model’s ability to distinguish between positive and negative samples. It is calculated as:


(17) $$MCC=\frac{TP\times TN-FP\times FN}{\left(TP+FP\right)\times \left(TP+FN\right)\times \left(TN+FP\right)\times \left(TN+FN\right)}$$

### 5.2 Comparisons with state-of-the-art methods

To assess the efficacy of our proposed CHMMI, we compared it with several widely adopted state-of-the-art image recognition algorithms. Specifically, we benchmarked our method against YOLOv5 Zhu et al. (2021), SSD Liu et al. (2016), Faster R-CNN Khan et al. (2022), ResNet He et al. (2016), FINet Zhang et al. (2022), YOLOT Liu et al. (2024), and an improved version of YOLOv5 (Improved_yolov5) Hu et al. (2024). These algorithms represent diverse architectural paradigms and have demonstrated exceptional performance across various computer vision tasks, providing a robust baseline for comparative analysis.

**Table 2** presents the quantitative results of the comparative analysis. As the table shows, our proposed CHMMI approach outperformed all the state-of-the-art methods across all four evaluation metrics. Specifically, CHMMI achieved an impressive AP of 0.841, surpassing the second-best performer, YOLOT, by a significant margin of 0.013. Furthermore, CHMMI attained the highest mAP@.5 of 0.887, outperforming the closest competitor, Improved_yolov5, by 0.006. CHMMI demonstrated its superiority in the most challenging mAP@.5:.95 metric, achieving a remarkable score of 0.551, 0.016 higher than the second-best performer, Improved yolov5. CHMMI performs excellently on the comprehensive evaluation metric MCC, achieving an outstanding score of 0.898, surpassing the second-place YOLOT by 0.011.

**Table 2:** Comparisons with state-of-the-art methods.

Method	AP	mAP@.5	mAP@.5:.95	MCC
YOLOv5 Zhu et al. (2021)	0.803	0.843	0.511	0.753
SSD Liu et al. (2016)	0.781	0.819	0.532	0.798
Faster R-CNN Khan et al. (2022)	0.629	0.757	0.521	0.647
ResNet He et al. (2016)	0.712	0.823	0.513	0.695
FINet Zhang et al. (2022)	0.637	0.869	0.524	0.823
YOLOT Liu et al. (2024)	0.828	0.877	0.531	0.887
Improved_yolov5 Hu et al. (2024)	0.807	0.881	0.535	0.873
**CHMMI**	**0.841**	**0.887**	**0.551**	**0.898**

**Figure 6:**
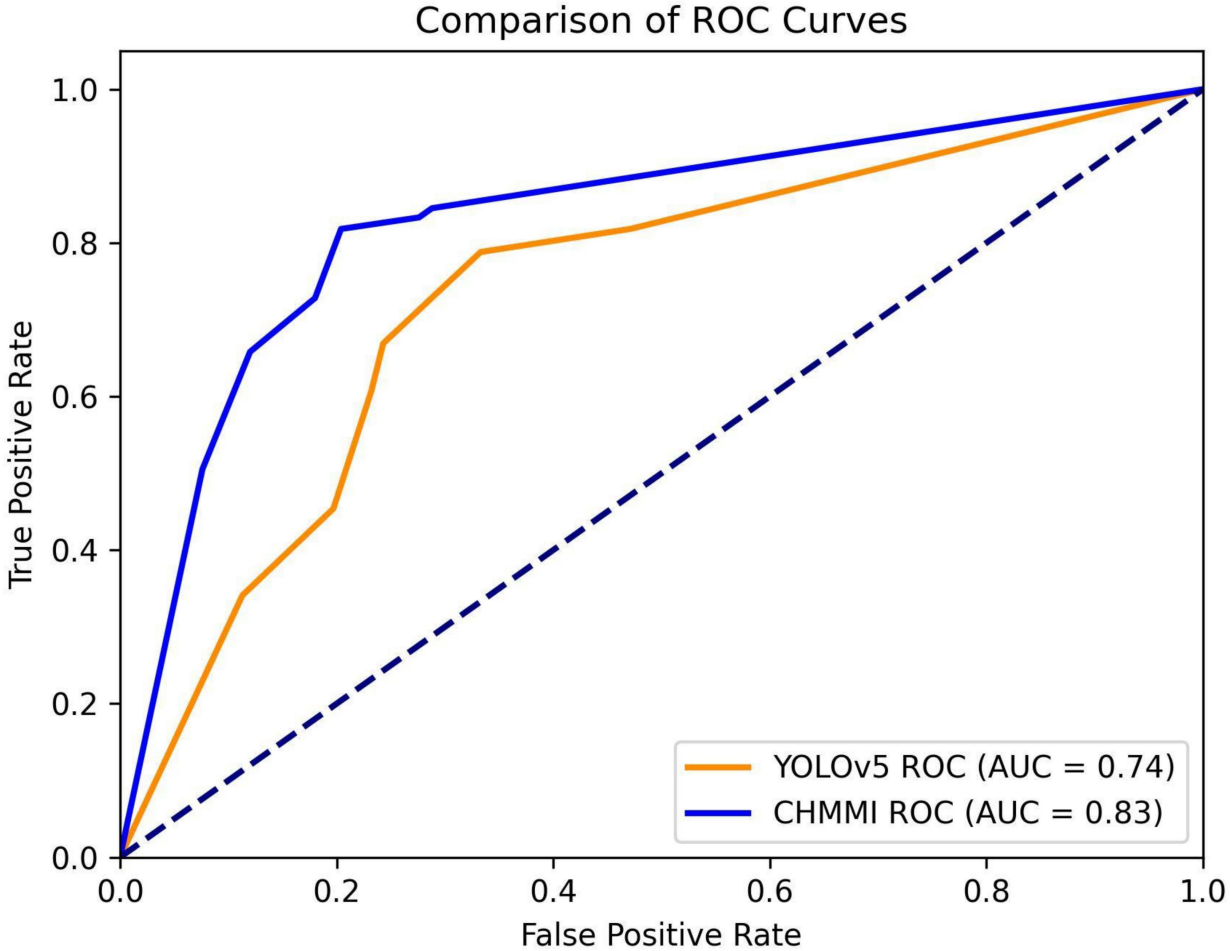
ROC Curves for models YOLOv5 and CHMMI.

To provide a visual representation of the performance difference, we plot the Receiver Operating Characteristic (ROC) curves for both YOLOv5 and CHMMI, using thresholds ranging from 0.1 to 1.0. **Figure 6** illustrates these curves, revealing a higher Area Under the Curve (AUC) value for CHMMI (0.83) compared to YOLOv5 (0.74), further confirming CHMMI’s superior performance.

In comparison to other CNN models, the CHMMI model has several advantages. For example, the YOLOv5 model uses a single-stage detection approach, which may not be suitable for handling the complexity of microscopic images. The SSD model uses a multi-scale feature fusion approach, but it may not be able to capture the contextual information of cells as effectively as the CHMMI model. The Faster R-CNN model uses a two-stage detection approach, but it may not be able to handle the issues of uneven cell distribution and incomplete and blurry cell structures as effectively as the CHMMI model. The ResNet model uses a residual learning approach, but it may not be able to capture the complex relationships between cells as effectively as the CHMMI model. These results underscore the efficacy of our proposed approach in accurately detecting and localizing objects under varying degrees of occlusion and overlap.

**Figure 7:**
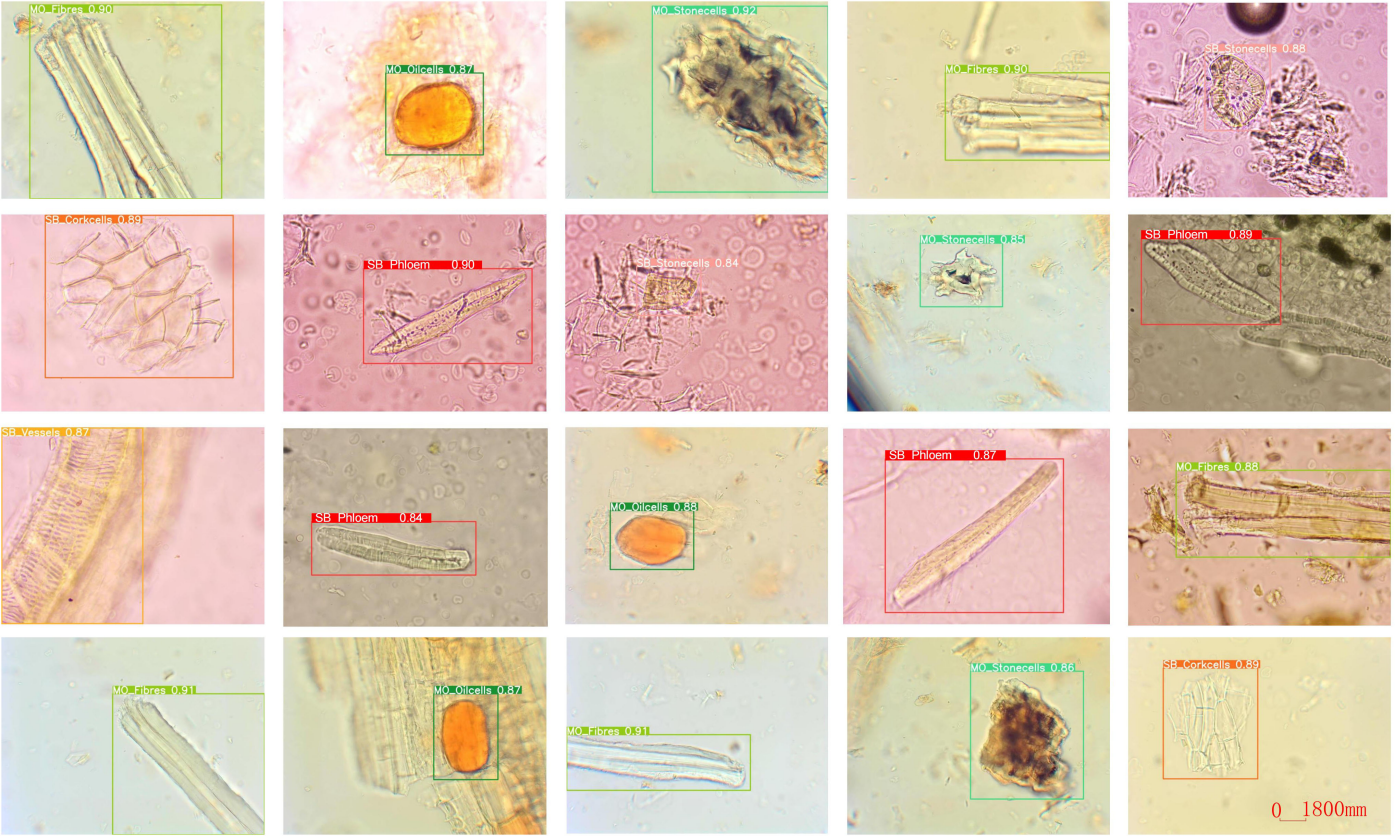
Visualization of the detection results using CHMMI.

In addition, we show the detection results of our CHMMI model, as shown in **Figure 7**. As can be seen from the figure, CHMMI can not only identify different categories of Chinese medicine feature cells but also accurately detect incomplete and blurriness cell structures.

### 5.3 Ablation studies

#### 5.3.1 Effectiveness of different modules

To assess the impact of each proposed module, we conducted a comprehensive set of ablation studies. Specifically, we systematically included or excluded the Microscopic Image Data Augmentation (MIDA), Shallow Channel Attention Module (SCAM), and Deep Channel Attention Module (DCAM) from our model and evaluated its performance. We employ a five-fold cross-validation strategy during the training phase to ensure a robust evaluation and mitigate the potential impact of data partitioning bias. The training dataset is divided into five non-overlapping subsets. For each fold, one subset is held out for validation, while the remaining four subsets are used for training. This process results in five distinct sets of model weights (M1, M2, M3, M4, and M5). During the testing phase, each of the five trained models (M1 to M5) is independently applied to the test set. This generates five sets of prediction results for each test sample. To combine these predictions, we implement a voting mechanism. The final predicted label for each test sample is determined by selecting the category that received the most votes across the five individual model predictions.

The data in **Table 3** demonstrates a clear trend of increasing model performance as more modules are incorporated. The inclusion of all three modules (MIDA, SCAM, and DCAM) results in the highest precision (P), recall(R), mAP@.5, and mAP@.5:.95. This suggests a synergistic effect between data augmentation, shallow feature attention, and deep feature attention mechanisms. The consistent improvement across all evaluation metrics indicates that the SDDA is vital in enhancing object detection accuracy. Furthermore, the results show that including MIDA alone significantly improves the model’s performance compared to using SCAM or DCAM individually. This highlights the importance of data augmentation in improving the model’s ability to detect objects in microscopic images. Integrating MIDA, SCAM, and DCAM leads to the most significant improvement in object detection accuracy, emphasizing the importance of combining data augmentation, shallow feature attention, and deep feature attention mechanisms.

**Table 3:** Experimental results using SCAM only, DCAM only, and SCAM+DCAM.

MIDA	SCAM	DCAM	Model	P	R	mAP@.5	mAP@.5:.95
			M1	0.874	0.789	0.834	0.507
			M2	0.804	0.809	0.845	0.508
			M3	0.811	0.763	0.839	0.509
			M4	0.821	0.787	0.812	0.491
			M5	0.793	0.830	0.844	0.511
			vote	0.831	0.808	0.843	0.511
			M1	0.883	0.791	0.842	0.520
			M2	0.845	0.811	0.864	0.522
✓			M3	0.841	0.793	0.840	0.512
			M4	0.833	0.801	0.818	0.497
			M5	0.799	0.837	0.858	0.517
			vote	0.854	0.835	0.855	0.522
			M1	0.884	0.811	0.869	0.521
			M2	0.821	0.809	0.863	0.518
	✓		M3	0.828	0.849	0.859	0.516
			M4	0.825	0.823	0.853	0.497
			M5	0.805	0.838	0.850	0.514
			vote	0.851	0.831	0.861	0.522
			M1	0.875	0.811	0.841	0.509
			M2	0.812	0.831	0.852	0.520
		✓	M3	0.859	0.798	0.855	0.530
			M4	0.825	0.812	0.847	0.493
			M5	0.831	0.846	0.860	0.517
			vote	0.856	0.835	0.868	0.528
			M1	0.891	0.825	0.878	0.530
			M2	0.859	0.815	0.867	0.522
✓	✓		M3	0.849	0.849	0.868	0.519
			M4	0.842	0.835	0.858	0.504
			M5	0.824	0.841	0.861	0.519
			vote	0.876	0.844	0.881	0.532
			M1	0.902	0.814	0.875	0.529
			M2	0.865	0.838	0.877	0.525
✓		✓	M3	0.864	0.823	0.857	0.535
			M4	0.838	0.826	0.850	0.506
			M5	0.835	0.847	0.869	0.527
			vote	0.868	0.845	0.879	0.537
			M1	0.893	0.821	0.877	0.524
			M2	0.863	0.848	0.861	0.538
	✓	✓	M3	0.866	0.848	0.860	0.537
			M4	0.858	0.839	0.849	0.518
			M5	0.845	0.857	0.865	0.532
			vote	0.873	0.854	0.879	0.541
			M1	0.917	0.838	0.885	0.531
			M2	0.897	0.850	0.875	0.549
✓	✓	✓	M3	0.874	0.856	0.883	0.546
			M4	0.871	0.849	0.866	0.534
			M5	0.861	0.871	0.882	0.543
			vote	0.905	0.871	0.887	0.551

The symbol ✓ indicates that the module has been selected.

#### 5.3.2 Effectiveness of microscopic image data augmentation module

The MIDA module plays a crucial role in enhancing the performance of our model by addressing the challenges posed by limited and imbalanced datasets of herbal microscopic images. It is particularly effective when dealing with images that only partially demonstrate certain features or cell types. By enhancing the representation of these specific attributes, we improve our data’s overall quality and diversity.

To evaluate the effectiveness of the MIDA module, we conducted extensive experiments by training our model with and without the augmented dataset generated by MIDA. The results, as shown in **Table 4**, demonstrate the significant impact of MIDA on the model’s performance metrics. As evident from the table, including the MIDA module resulted in significant improvements across all performance metrics. The precision and recall values increased from 0.831 and 0.808, respectively, without MIDA to 0.854 and 0.835 with MIDA, indicating a substantial enhancement in the model’s ability to accurately classify cell types and features while minimizing false positives and false negatives. Moreover, the mean Average Precision (mAP) values, which comprehensively evaluate the model’s performance across different confidence thresholds, also exhibited notable improvements. The mAP@.5, which measures the average precision at an intersection-over-union (IoU) threshold of 0.5, increased from 0.843 without MIDA to 0.855 with MIDA. Similarly, the mAP@.5:.95, which averages the precision values across IoU thresholds ranging from 0.5 to 0.95, improved from 0.511 to 0.522 with MIDA.

**Table 4:** Experimental results with and without microscopic image data augmentation module.

Module Name	P	R	mAP@.5	mAP@.5:.95
**w/o MIDA**	0.831	0.808	0.843	0.511
**w MIDA**	0.854	0.835	0.855	0.522

#### 5.3.3 Effectiveness of shallow-deep dual attention module

**Figure 8:**
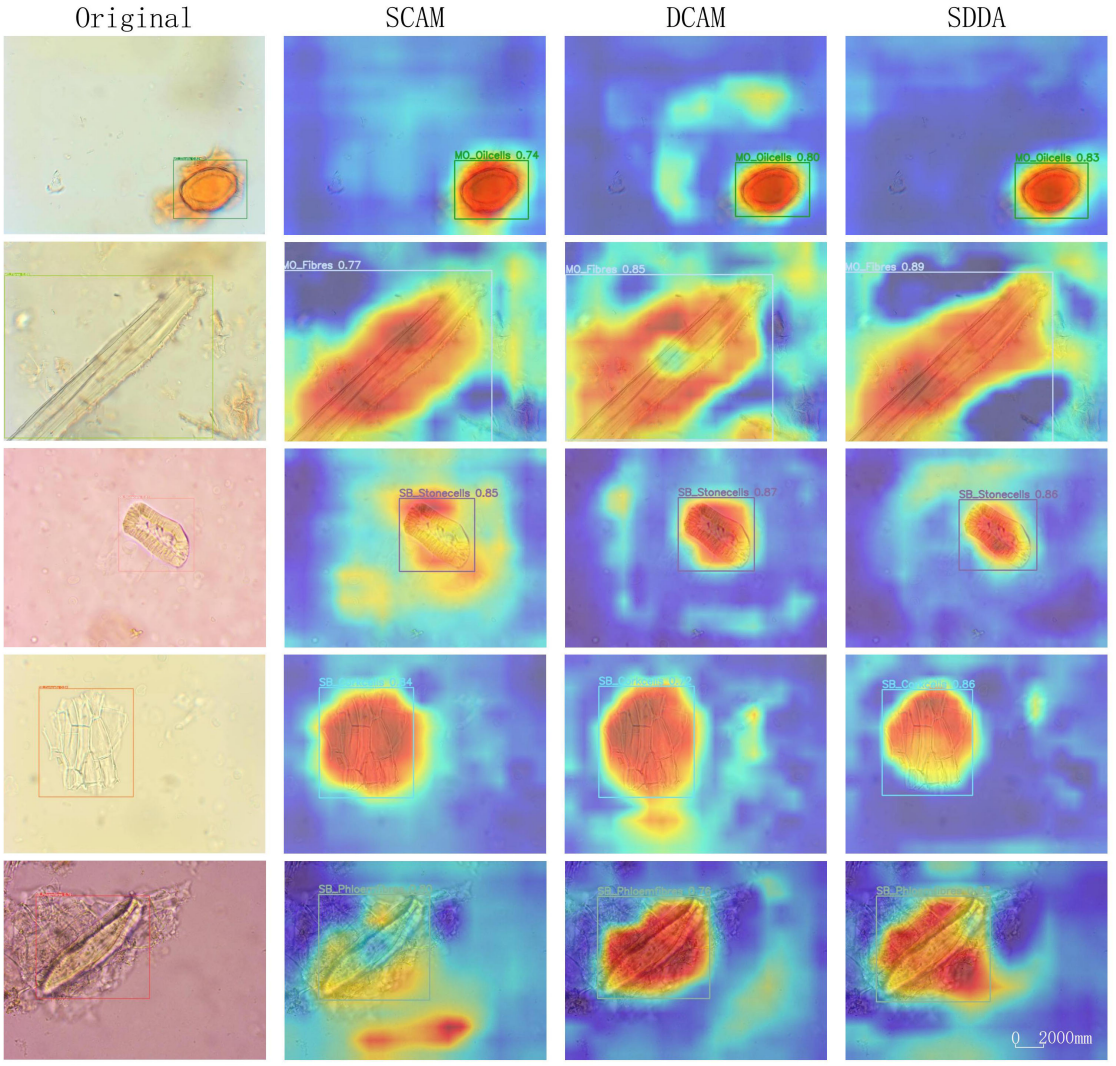
Heatmap examples using SCAM only, DCAM only, and SDDA.

The SDDA module represents a significant advancement in addressing the complex challenges inherent in the microscopic examination of CHM cells. This module integrates the strengths of both shallow and deep feature representations within the model. The heatmaps in **Figure 8** provide a visual representation of the impact of the SDDA module. When only the SCAM is used, the model tends to focus on less relevant areas, potentially discarding crucial feature information. Conversely, when only the DCAM is used, the attention becomes scattered, hindering the model’s ability to focus on the foreground regions of interest precisely. However, the simultaneous use of both SCAM and DCAM results in a focused and accurate attention map, highlighting the model’s ability to detect cells with diverse morphological features, even incomplete or blurry.

Overall, the Shallow-Deep Dual Attention module effectively enhances the CHMMI model’s ability to accurately detect and analyze CHM cells by addressing the limitations of individual attention mechanisms. The combination of SCAM and DCAM allows the model to focus on relevant features and handle various challenges in microscopic cell examination, leading to improved performance and more accurate results.

## 6 Conclusion

Traditional Chinese Herbal Medicine (CHM) identification methodologies, such as original plant identification, character identification, microscopic identification, and physical and chemical identification, have long been relied upon but present significant challenges regarding labor intensity, subjectivity, and limitations in distinguishing similar substances. The rapid growth of the CHM market and the need for modernization call for more advanced and reliable identification techniques. Developing deep learning-based methods, particularly artificial neural networks, offers a promising solution to automate CHM microscopic identification. Our proposed methodology, CHMMI, addresses key challenges in automated CHM identification by combining segmentation methods with data augmentation and integrating attention mechanisms to enhance feature recognition and model accuracy. By effectively capturing small and uneven features and addressing issues with incomplete and blurry cell structures in CHM samples, CHMMI outperforms existing state-of-the-art approaches in experimental comparisons. CHMMI can be integrated into the quality control processes of CHM manufacturers. Automating the identification of herbal components can ensure consistency in raw material selection, detect adulterants or contaminants, and maintain the purity of herbal preparations. This application could significantly improve product quality and safety, potentially reducing the risk of adverse reactions due to misidentified or contaminated herbs. CHMMI can accelerate the discovery of new bioactive compounds from traditional herbal medicines in pharmaceutical research. By quickly and accurately identifying cellular structures, researchers can more efficiently screen large numbers of herbal samples, potentially leading to the development of novel drugs or therapies.

While CHMMI shows superior performance, understanding why certain features are prioritized over others could be beneficial. Future research will focus on developing or integrating explainable AI techniques to provide insights into the model’s decision-making process, enhancing trust and acceptance in clinical and regulatory settings.

## Data Availability

The data analyzed in this study was obtained from Wuzhou University and Guangxi Wuzhou Zhongheng Group Co., Ltd. The following licenses/restrictions apply: Data is available for academic research use only and may not be redistributed or used for commercial purposes without prior approval. Requests to access these datasets should be directed to Dr Guangyao Pang via email at pangguangyao@gmail.com.
